# Assessments of the In Vitro and In Vivo Linker Stability and Catabolic Fate for the Ortho Hydroxy-Protected Aryl Sulfate Linker by Immuno-Affinity Capture Liquid Chromatography Quadrupole Time-of-Flight Mass Spectrometric Assay

**DOI:** 10.3390/pharmaceutics13010125

**Published:** 2021-01-19

**Authors:** Byeong ill Lee, Seo-jin Park, Yuri Park, Seok-Ho Shin, Jang-mi Choi, Min-jae Park, Jeong-hyeon Lim, Sun Young Kim, Hyangsook Lee, Young G. Shin

**Affiliations:** 1 College of Pharmacy and Institute of Drug Research and Development, Chungnam National University, Daejeon 34134, Korea; byungill.lee.cnu@gmail.com (B.i.L.); seojin.park.cnu@gmail.com (S.-j.P.); yuri.park.cnu@gmail.com (Y.P.); seokho.shin.cnu@gmail.com (S.-H.S.); jangmi.choi.cnu@gmail.com (J.-m.C.); minjae.park.cnu@gmail.com (M.-j.P.); jeonghyeon.lim.cnu@gmail.com (J.-h.L.); 2 IntoCell Inc., 101, Sinildong-ro, Daedeok-gu, Daejeon 34324, Korea; sykim@intocell.co.kr (S.Y.K.); hslee@intocell.co.kr (H.L.)

**Keywords:** antibody–drug conjugate, LC-qTOF-MS, OHPAS linker, VC-PABC linker, linker stability, catabolic fate

## Abstract

Antibody–drug conjugate (ADC) linkers play an important role in determining the safety and efficacy of ADC. The Ortho Hydroxy-Protected Aryl Sulfate (OHPAS) linker is a newly developed linker in the form of a di-aryl sulfate structure consisting of phenolic payload and self-immolative group (SIG). In this study, using two bioanalytical approaches (namely “bottom-up” and “middle-up” approaches) via the liquid chromatography-quadrupole time-of-flight mass spectrometric (LC-qTOF-MS) method, in vitro and in vivo linker stability experiments were conducted for the OHPAS linker. For comparison, the valine-citrulline-*p*-aminobenzyloxycarbonyl (VC-PABC) linker was also evaluated under the same experimental conditions. In addition, the catabolite identification experiments at the subunit intact protein level were simultaneously performed to evaluate the catabolic fate of ADCs. As a result, the OHPAS linker was stable in the in vitro mouse/human plasma as well as in vivo pharmacokinetic studies in mice, whereas the VC-PABC linker was relatively unstable in mice in vitro and in vivo. This is because the VC-PABC linker was sensitive to a hydrolytic enzyme called carboxylesterase 1c (Ces1c) in mouse plasma. In conclusion, the OHPAS linker appears to be a good linker for ADC, and further experiments would be warranted to demonstrate the efficacy and toxicity related to the OHPAS linker.

## 1. Introduction

The antibody–drug conjugate (ADC) is a targeted anti-cancer agent consisting of a monoclonal antibody, linkers, and cytotoxic drugs (payload) [1,2,3,4,5,6]. The major characteristics of ADCs for anti-cancer therapy are that it can improve the therapeutic window by delivering cytotoxic drugs selectively to target cells [7,8,9]. Therefore, ADC is expected to solve unmet needs that cannot be solved with existing anti-cancer therapy. Since Mylotarg^®^ (gemtuzumab ozogamicin) was approved as the first ADC in 2000, nine ADCs have been approved to date, of which five ADCs have been approved in the last two years. In addition, approximately 70 ADCs are undergoing clinical trials, and many more ADCs are under preclinical developments [9,10,11]. Over 30 years of intensive research and development, activities have made many advances in antibody engineering, linker conjugation platforms, and the diversity of new payloads. In particular, the linker conjugation platform plays an important role in determining the drug–antibody ratio (DAR) complexity and the stability of ADCs in systemic circulation. If the linker was unstable during systemic circulation, ADC’s mechanism of action in the target cell would be lost, and the deconjugated linker-payload would be able to cause off-target toxicity by adducting to other proteins or metabolizing to other forms [12,13,14,15,16]. Therefore, many pharmaceutical companies have intensively researched and developed various linker conjugation platforms [16,17,18,19].

Recently, the Ortho Hydroxy-Protected Aryl Sulfate (OHPAS) linker was developed as a new linker platform by IntoCell Inc. (Daejeon, Korea) and structurally designed with di-aryl sulfate, in which one aryl acting as a phenolic payload and the other acting as a self-immolative group (SIG) consisting a triggering phenol function at the ortho or para position. In the model study with tyrosine substrate of the previous reference, the OHPAS linker was stable in various species plasma and was able to release the payloads when triggered by beta-galactosidase, which is highly expressed in tumor cells [20,21,22].

**Figure 1:**
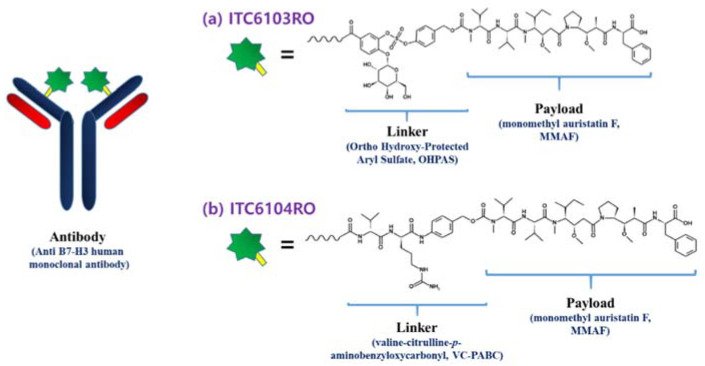
Structure of (**a**) ITC6103RO and (**b**) ITC6104RO.

In this study, the linker stability of two ADCs with the OHPAS linker and the valine-citrulline-*p*-aminobenzyloxycarbonyl (VC-PABC) linker, which has been widely applied as SIG chemistry by the ADC research group [23], was evaluated using the immuno-affinity capture liquid chromatography-quadrupole time-of-flight mass spectrometric (LC-qTOF-MS) assays. The two ADCs are ITC6103RO (consisting of B7-H3-targeting human monoclonal antibody, OHPAS linker and monomethyl auristatin F (MMAF)) and ITC6104RO (consisting of B7-H3-targeting human monoclonal antibody, VC-PABC linker and MMAF), respectively, and are shown in Figure 1. For both ADCs, the linkers and payloads were site-specifically conjugated to the cysteine residues in the Fd’ region of monoclonal antibody by applying the THIOMAB™ antibody technology platform. Fd’ region corresponds to the antigen-binding fragment (Fab) region of the N-terminal heavy chain of antibody. Therefore, the DAR value of both ADCs was 2.

In general, there are three bioanalytical approaches to measure components of ADC using the immuno-affinity capture LC-qTOF-MS method: “top-down”, “middle-up” and “bottom-up” approaches [24,25,26]. Among them, the “top-down” approach has the advantage of being able to measure unmodified intact ADCs without any treatment such as enzymatic digestion, and thus can provide structural information of the ADC. However, this approach has limitation to the analysis of bioanalytical samples at low concentration levels particularly in vivo pharmacokinetic (PK) study [13,27,28,29]. To overcome these shortcomings of the “top-down” approach, the “middle-up” approach, which is an intact protein mass analysis through partial enzymatic digestion and chemical reduction, has also been developed to provide information about the regions of structural interest of the ADC in bioanalytical samples. The FabRICATOR^®^ (known as IdeS) is a cysteine protease that digests antibodies at specific sites under the hinge, and is mainly used for partial digestion. The “bottom-up” approach is a bioanalytical method in which antibodies are proteolyzed into a primary sequence peptide using a proteolytic enzyme such as trypsin [25,26,30,31]. Using this approach, it is possible to quantify total antibody (tAb) through the determination of a signature peptide and also to perform peptide mapping analysis. In the case of ADC with cleavable linkers, it is also possible to quantify antibody-conjugated drug (acDrug) by the usage of linker-specific enzyme such as cathepsin B, papain and beta-galactosidase [4,5].

In this work, the in vitro and in vivo linker stability was evaluated using “middle-up” and “bottom-up” approaches for the components of ADC as well as the DAR profile from in vitro mouse and immunoglobulin G (IgG) depleted human plasma and in vivo mouse preclinical pharmacokinetic studies. In the “bottom-up” approach, concentrations of a specific signature peptide present only in the Fc region of human monoclonal antibody (representative amounts of tAb) by trypsin digestion as well as the acDrug released by linker site-specific cleavable enzyme (representative amounts of ADC) were quantified, and then the DAR profiles of ADCs were further calculated by the relative ratio of the two components in molar concentrations (acDrug vs. tAb). In the “middle-up” approach, a partial digestion by FabRICATOR^®^ and reduction by dithiothreitol (DTT) were performed to measure the intensities of the light chain and the cytotoxic drug-conjugated Fd’ region (Fd’ + DAR 1), and then the DAR profiles were calculated by the relative intensity ratio of the two components (Fd’ + DAR 1/light chain). In addition, using the “middle-up” approach, catabolite identification studies at the linker conjugation site were performed simultaneously to determine the metabolites/catabolites during the DAR profile changes.

## 2. Materials and Methods

### 2.1. Chemicals and Reagents

ITC6103RO, ITC6104RO and MMAF were obtained from IntoCell Inc. (Daejeon, Korea). The stable isotope-labeled peptide (TTPP*V*LDSDGSFFLYSK, * indicates a stable isotope labeled amino acid with 5 carbons (^12^C to ^13^C) and 1 nitrogen (^14^N to ^15^N)) which was used for the internal standard (ISTD1) for total antibody quantification assay was purchased from AnyGen (Jeollamnam-do, Korea). Verapamil, which was used for another internal standard (ISTD2) for acDrug and free payload quantification assay, and β-galactosidase and papain were purchased from Sigma-Aldrich Korea (Seoul, Korea).

FabRICATOR^®^ was purchased from Genovis (Lund, Sweden). The protein A magnetic bead was purchased from Millipore Korea (Daejeon, Korea), and the protein L magnetic bead was purchased from Bioclone (Seoul, Korea). The sequencing-grade modified trypsin was purchased from Promega (Madison, WI, USA). RapiGest SF^®^ was purchased from Waters Korea (Seoul, Korea), and DTT was purchased from Carl Roth (Karlsruhe, Germany). Iodoacetic acid (IAA) was purchased from Wako (Osaka, Japan). All other chemicals were commercial products of analytical or reagent grade and used without further purification.

### 2.2. Sample Preparation Methods for Quantification of tAb, acDrug and Free Payload

#### 2.2.1. Preparation of Calibration Standards, Internal Standards (ISTD) and Quality Control (QC) Samples

Stock solutions (10 mg/mL) of ITC6103RO and ITC6104RO were prepared in PBS and stored at 4 °C. After that, sub-stock solutions with 0.5 mg/mL were made by diluting the stock solution using 0.1% tween in PBS. Stock solutions (1 mg/mL) of MMAF were prepared in dimethyl sulfoxide (DMSO) and stored at −20 °C. After that, sub-stock solutions with 0.1 mg/mL were made by diluting the stock solution using DMSO.

For tAb quantification assay, calibration standard samples were made at concentrations of 0.5, 1, 2, 5, 10, 20, 40, 80 and 100 μg/mL in blank plasma. Three levels of QC samples were also made at 2.5 (QC low), 25 (QC medium) and 50 μg/mL (QC high) with blank plasma. The ISTD1 solution was prepared at a concentration of 10 μg/mL in PBS using a stable isotope-labeled peptide. For acDrug quantification assay, calibration standard samples were made at concentrations of 0.98, 4.88, 9.76, 19.5, 48.8, 97.7, 195.3, 390.7, 781.3 and 976.7 ng/mL in blank plasma. Three levels of QC samples were also made at 24.4 (QC low), 244.2 (QC medium) and 488.3 ng/mL (QC high) with blank plasma. For free payload quantification assay, calibration standard samples were made at concentrations of 1.01, 3.02, 9.05, 27.2, 81.5, 244, 733 and 2200 ng/mL in blank plasma. Three levels of QC samples were also made at 15 (QC low), 165 (QC medium) and 1820 ng/mL (QC high) with blank plasma. The ISTD2 spiking solution containing 100 ng/mL of verapamil was prepared in acetonitrile. 

#### 2.2.2. Total Antibody (tAb) Quantification Assay by Trypsin Digestion

Each 20 μL aliquot of the calibration curve, QC and study samples was mixed with 350 μL of 0.1% tween in PBS and 30 μL of protein A magnetic bead suspension. After gently shaking at room temperature for 2 h, protein A magnetic beads were washed once with 200 μL of 0.1% tween in PBS and then washed again with 200 μL of PBS. A 25 μL aliquot of ISTD1 solution, 75 μL of RapiGest and 10 μL of 0.1 M DTT were mixed with washed magnetic bead samples to denature and reduce antibodies of ADC. After shaking for 1 min, the samples were incubated for 1 h at 60 °C. Then, 25 μL of IAA was added to the samples to alkylate antibodies of ADC. After shaking for another 1 min, the samples were incubated for 30 min at room temperature. A 10 μL aliquot of the sequencing-grade modified trypsin was added to the samples for enzymatic digestion. After shaking for 1 min, the samples were incubated at 37 °C overnight. A 15 μL aliquot of 2 M hydrochloric acid (HCl) was added to the samples to quench the reaction. After shaking for 1 min, the samples were incubated for 30 min at 37 °C. The samples were then centrifuged at 10,000 rpm for 5 min, and the supernatants were transferred into an LC vial for LC-qTOF-MS analysis. 

#### 2.2.3. Antibody-Conjugated Drug (acDrug) Quantification Assay by Linker Cleavable Enzymes Digestion

Each 20 μL aliquot of the calibration curve, QC and study samples was mixed with 350 μL of 0.1% tween in PBS and 20 μL of protein A magnetic bead suspension. After gently shaking at room temperature for 2 h, protein A magnetic beads were washed once with 200 μL of 0.1% tween in PBS and then washed again with 200 μL of PBS. For acDrug quantification of ITC6103RO, 40 μL of β-galactosidase was added to the washed magnetic bead samples to release acDrug from ADC. For acDrug quantification of ITC6104RO, 60 μL of papain (0.2 mg/mL) in 10 mM ethylenediaminetetraacetic acid (EDTA) was added to the washed magnetic bead samples to release acDrug from ADC. After shaking for 1 min, the samples were incubated at 37 °C overnight. A 40 μL aliquot of ISTD2 spiking solution was added to the samples to quench the reaction. After shaking for 1 min, the samples were then centrifuged at 10,000 rpm for 5 min and the supernatants were transferred into an LC vial for LC-qTOF-MS analysis.

#### 2.2.4. Free Payload Quantification Assay

A 100 μL aliquot of ISTD2 spiking solution was added to a 20 μL aliquot of calibration curve, QC and study samples for protein precipitation. After shaking this mixture for 1 min, the samples were centrifuged at 10,000 rpm for 5 min. Following the centrifugation, 50 µL of supernatant samples was transferred to another tube and diluted by adding 100 µL of DW. Then, the mixture samples were transferred to an LC vial for LC-qTOF-MS analysis.

### 2.3. Sample Preparation Methods for Intact Protein Mass Analysis

Each 100 μL aliquot of the study samples was mixed with 350 μL of 0.1% tween 20 in PBS and 20 μL of protein L magnetic bead suspension. After gently shaking at room temperature for 2 h, the protein L magnetic beads were washed once with 200 μL of 0.1% tween in PBS and then washed again with 200 μL of PBS. Amounts of 100 μL of PBS and 2 μL of FabRICATOR^®^ were added to the washed magnetic bead samples for partially digestion to generating F(ab’)2 and Fc/2 fragments from ADC. After shaking for 1 min, the samples were incubated for 30 min at 37 °C. After removing supernatants, a 50 μL aliquot of PBS and 2 μL of 0.5M DTT were added to the samples to reduce F(ab’)2 fragments. After shaking for 1 min, the samples were incubated for 40 min at 37 °C. Then, a 50 μL aliquot of 1% formic acid in 30% acetonitrile spiking solution was added to the samples to quench the reaction. After shaking for 1 min, the samples were then centrifuged at 10,000 rpm for 5 min and the supernatants were transferred into an LC vial for LC-qTOF-MS analysis.

### 2.4. Application for In Vitro Linker Stability Studies

For the in vitro linker stability test, stock solutions of ITC6103RO and ITC6104RO were spiked into the blank ICR mouse plasma (BioMedex Korea, Seoul, Korea) and the IgG depleted human plasma (BioMedex Korea, Seoul, Korea) to make incubation samples at the concentration of 90 μg/mL. The samples were incubated in 37 °C for 0, 1, 3, 5 and 7 days. After incubation, the samples were stored at −80 °C until analysis.

### 2.5. Application for Preclinical Pharmacokinetic Studies in Mice

The preclinical PK studies were conducted in ICR mice. ITC6103RO and ITC6104RO were administered to mice via single intravenous bolus injection (3 mg/kg). The blood sampling times were 0, 0.002, 0.042, 0.167, 0.29, 1, 2, 4, 7, 14, 21 and 28 days, and each of the blood samples was collected in heparinized tubes. The blood samples were centrifuged, and the supernatant plasma samples were stored at −80 °C until analysis. Animal experiments followed the animal care protocol (no. 202003A-CNU-023) approved by the Chungnam National University on 1 April 2020. All procedures related to animal experiments were also performed in accordance with the guidelines established by the Association for Assessment and Accreditation of Laboratory Animal Care International (AAALAC International).

### 2.6. LC-MS Conditions

The liquid chromatography–high resolution mass spectrometric system consisted of two Shimadzu LC-20 AD pumps, a Shimadzu CBM-20A HPLC pump controller (Shimadzu Corporation, Columbia, MD, USA), a CTC HTS PAL autosampler (LEAP Technologies, Carrboro, NC, USA) and a quadrupole time-of-flight TripleTOF™ 5600 mass spectrometer (Sciex, Foster City, CA, USA).

For bioanalytical sample quantification, a HPLC analytical column was Phenomenex Kinetex^®^ XB–C18 column (2.1 × 50 mm, 2.6 μm). The mobile phase consisted of mobile phase A, 0.1% formic acid in distilled water, and mobile phase B, 0.1% formic acid in acetonitrile. The gradient was delivered at a flow rate of 0.4 mL/min. The gradient used for tAb quantification assay was as follows: from 0 to 0.5 min, 15% B; from 0.5 to 1.4 min by a linear gradient from 15 to 70% B; from 1.4 to 1.5 min by a linear gradient from 70 to 95% B; 95% B was maintained for 0.2 min; from 1.7 to 1.8 min by a linear gradient from 95 to 15% B, and then 15% B was maintained for 1.2 min. The gradient used for acDrug and free payload quantification assay was as follows: from 0 to 0.6 min, 10% B; from 0.6 to 1.0 min by a linear gradient from 10 to 95% B; 95% B was maintained for 0.3 min; from 1.3 to 1.4 min by a linear gradient from 95 to 10% B, and then 10% B was maintained for 1.6 min. 

For intact protein mass analysis, HPLC analytical column was Phenomenex bioZen™ WidePore C4 (2.1 × 50 mm, 2.6 μm). The mobile phase consisted of mobile phase A, 0.1% formic acid in distilled water, and mobile phase B, 0.1% formic acid in 90% acetonitrile. The gradient was delivered at a flow rate of 0.4 mL/min. The gradient was as follows: from 0 to 0.5 min, 10% B; from 0.5 to 4.5 min by a linear gradient from 10 to 90% B; 90% B was maintained for 0.3 min; from 4.8 to 4.9 min by a linear gradient from 90 to 10% B, and then 10% B was maintained for 1.1 min. 

The TOF-MS scan mass spectra and product ion scan mass spectra were recorded in positive ion mode with a resolution of ~35,000 full-width half-maximum and a mass accuracy of <5 ppm. The source gas (nebulizer (GS1) and heater (GS2)) were set at 50 psi, and the source temperature was set at 500 °C with the curtain gas (CUR) flow of 33 L/min. The ion spray voltage (ISVF) was set at 5000 and 5500 V for intact protein mass analysis and quantification assay, respectively. Other mass spectrometric conditions are summarized in Table 1 and Table 2.

### 2.7. Softwares

An LC-qTOF-MS operation and data acquisition were conducted using Analyst^®^ TF version 1.6 (Sciex, Foster City, CA, USA). Data processing was conducted using MultiQuant^®^ version 3.0.3 (Sciex, Foster City, CA, USA) for quantification and PeakView^®^ version 2.2 (Sciex, Foster City, CA, USA), BiopharmaView™ version 3.0 (Sciex, Foster City, CA, USA) for intact protein mass analysis data processing. Graph visualization was performed using GraphPad Prism version 8 (GraphPad Software Inc., San Diego, CA, USA). Chemical structures were drawn using Medchem Designer version 5.0 (Simulations Plus, Lancaster, CA, USA). Pharmacokinetic parameters were calculated using non-compartmental analysis (NCA) and a two-compartment model with Phoenix WinNonLin^®^ version 8.0.0 (Certara, Princeton, NJ, USA).

## 3. Results and Discussion

### 3.1. In Vitro Linker Stability in Mouse Plasma and IgG Depleted Human Plasma

**Figure 2:**
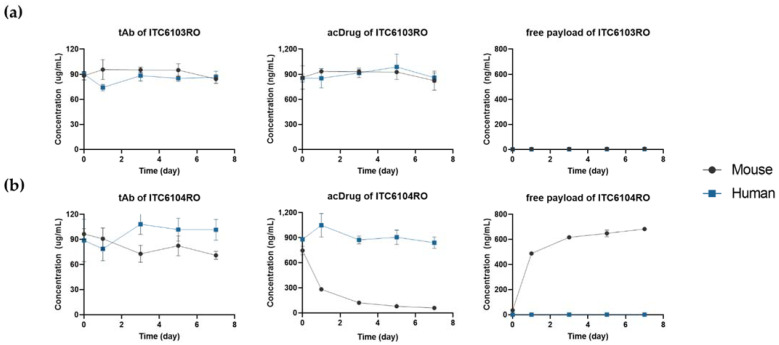
Time-concentration profiles of total antibody (tAb), antibody-conjugated drug (acDrug) and free payload for (**a**) ITC6103RO and (**b**) ITC6104RO from in vitro linker stability in mouse and IgG depleted human plasma.

The in vitro linker stability plasma samples for ITC6103RO and ITC6104RO were analyzed by using the LC-qTOF-MS assays. The time-concentration profiles for tAb, acDrug and free payload of ITC6103RO and ITC6104RO were calculated and are shown in Figure 2. 

**Figure 3:**
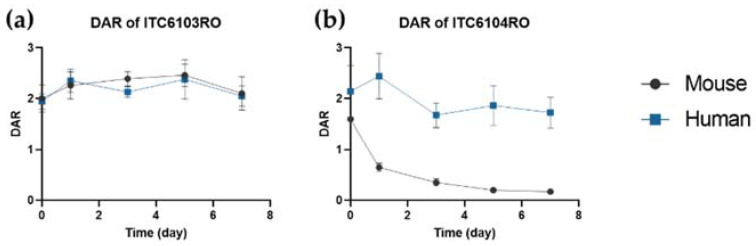
The drug–antibody ratio (DAR) profiles from in vitro linker stability in mouse and IgG depleted human plasma for (**a**) ITC6103RO and (**b**) ITC6104RO.

Figure 2 showed that both tAb and acDrug of ITC6103RO were stable in mouse and IgG depleted human plasma, whereas in ITC6104RO, only tAb was stable in mouse plasma, not acDrug. Due to instability of acDrug, the concentration of free payload for ITC6104RO also gradually increased for 7 days. The amount of free payload released on the 7th day reached almost 80% when compared to the amount of initial acDrug. The DAR profiles were calculated by the following Equation (1) and are shown in Figure 3.

(1) $$\mathrm{DAR}=\left(\frac{\frac{\mathrm{concentration}\;\mathrm{of}\;\mathrm{acDrug}\;\left(\mathrm{ng}/\mathrm{mL}\right)}{\mathrm{molecular}\;\mathrm{weight}\;\mathrm{of}\;\mathrm{payload}\;\left(\mathrm{MMAF}\;=\;731.9\;\mathrm{Da}\right)}}{\frac{\mathrm{concentration}\;\mathrm{of}\;\mathrm{tAb}\;\left(µg/\mathrm{mL}\right)}{\mathrm{molarcular}\;\mathrm{weight}\;\mathrm{of}\;\mathrm{ADC}\;\left(\mathrm{about}\;150\;\mathrm{kDa}\right)}}\right)$$

The DAR profile of ITC6104RO gradually decreased in mouse plasma, which was likely due to the instability of the VC-PABC linker by the carboxylesterase 1 (Ces1c) in mouse plasma, which was reported in previous references [32,33].

### 3.2. In Vivo Pharmacokinetic Study in Mice

**Figure 4:**
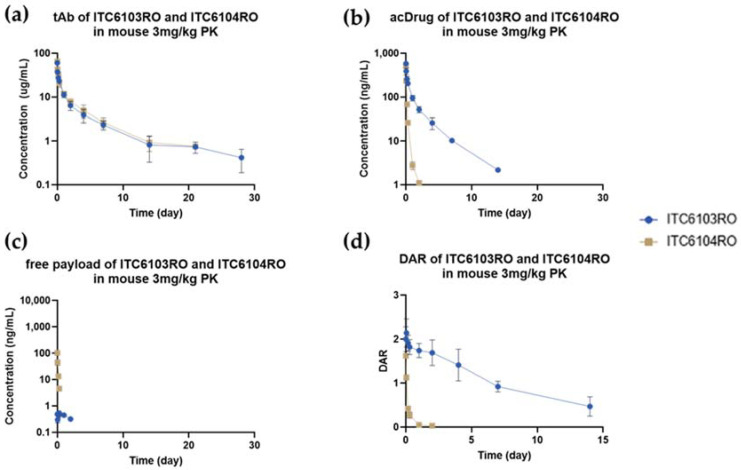
Pharmacokinetic (PK) profiles of (**a**) tAb, (**b**) acDrug, (**c**) free payload and the (**d**) calculated DAR profiles after intravenous administration of 3 mg/kg for ITC6103RO and ITC6104RO, respectively, in mice.

The in vivo mouse plasma samples obtained after intravenous administration of 3 mg/kg of ITC6103RO and ITC6104RO were also analyzed by the LC-qTOF-MS assays. The pharmacokinetic profiles of tAb, acDrug and free payload and the calculated DAR profiles for ITC6103RO and ITC6104RO are shown in Figure 4.

Similar to the in vitro linker stability in mouse plasma, the acDrug concentration decreased rapidly, and so the DAR profile also decreased for ITC6104RO ADC with the VC-PABC linker. The PK parameters for tAb and acDrug are shown in Table 3 and Table 4, respectively. 

From the PK perspective, there was no significant difference in tAb concentrations between ITC6103RO and ITC6104RO. However, in the case of acDrug, ITC6104RO showed much lower exposure and higher clearance than ITC6103RO. Therefore, the OHPAS linker of ITC6103RO appears to be more stable than the VC-PABC linker of ITC6104RO in the systemic circulation in mice.

### 3.3. Intact Protein Mass Analysis by Partial Digestion and Reduction

**Figure 5:**
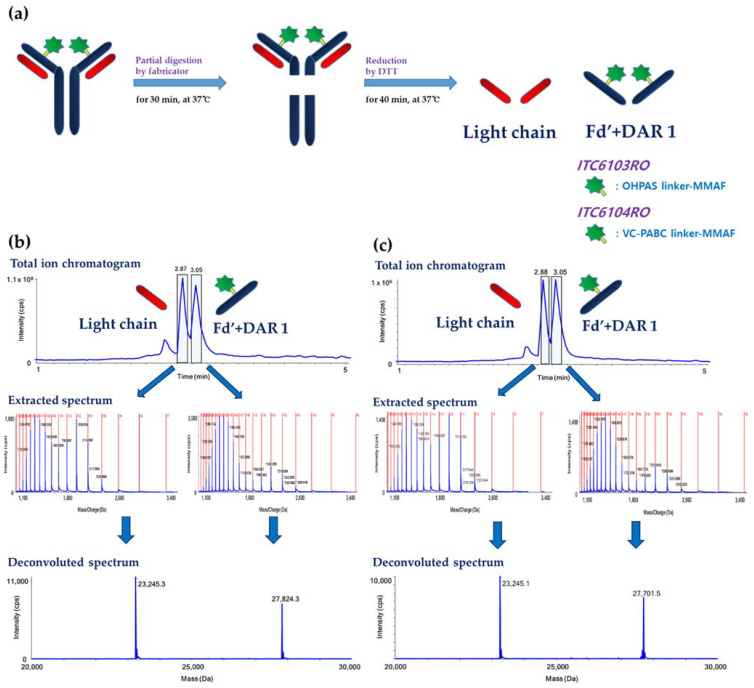
(**a**) Workflow of intact ADC mass analysis at subunit intact protein level by partial digestion and reduction, and the representative total ion chromatogram (TIC) with the extracted and the deconvoluted spectra of the quality control sample for (**b**) ITC6103RO and (**c**) ITC6104RO.

For the “middle-up” approach via LC-qTOF-MS methods, the light chain and Fd’ + DAR 1 regions of both ADCs were chromatographically separated from the endogenous mouse immunoglobulin, and the intensities of each region were calculated through the workflow shown in Figure 5.

**Figure 6:**
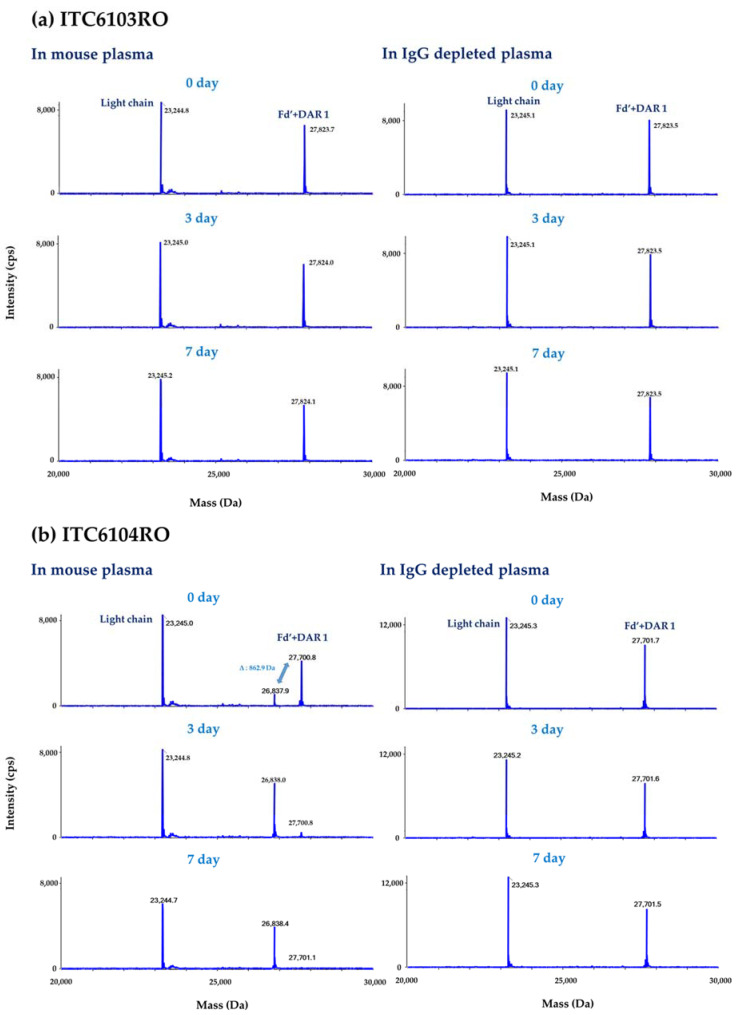
The deconvoluted spectra for (**a**) ITC6103RO and (**b**) ITC6104RO from the in vitro linker stability in mouse plasma and the IgG depleted human plasma samples.

**Figure 7:**
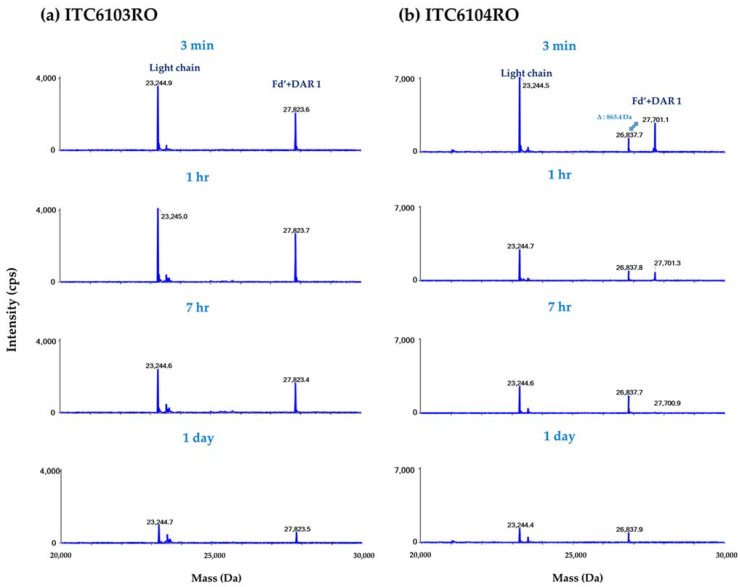
The deconvoluted spectra for (**a**) ITC6103RO and (**b**) ITC6104RO from the in vivo mouse pharmacokinetic samples.

According to the aforementioned workflow, both the in vitro linker stability samples and the in vivo pharmacokinetic study samples were analyzed in each species plasma, and the results are shown in Figure 6 and Figure 7, respectively.

For ITC6103RO, no significant peak was detected except for the light chain and Fd’ + DAR 1 from all in vitro linker stability samples and the in vivo mouse pharmacokinetic samples. On the other hand, for ITC6104RO, the intensity of the Fd’ + DAR 1 gradually decreased over time for in vitro and in vivo mouse samples, and the intensity of an unknown peak with a molecular weight of 26,837.9 Da (molecular weight of Fd’ + DAR 1 − 862.9 Da) increased. Based on the molecular weight and the mass difference, this is considered to be a catabolite of the VC-PABC linker by carboxylesterase 1 (Ces1c) in mouse plasma.

For the DAR profile analysis, the relative ratio of intensities between the light chain and Fd’ + DAR 1 at 0 day was set as 100%, and the relative ratio of intensities over time was expressed as the remaining %. The remaining % of the DAR was calculated by the “middle-up” approach and was also compared to the values by the “bottom-up” approach.

**Figure 8:**
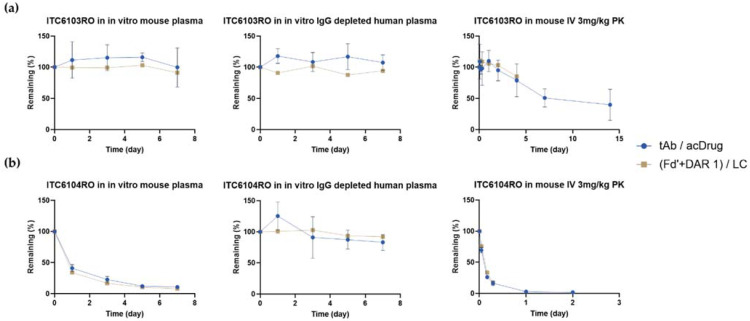
DAR profiles for (**a**) ITC6103RO and (**b**) ITC6104RO from the in vitro linker stability and the in vivo mouse pharmacokinetic samples, respectively.

As shown in Figure 8, there was no significant difference in terms of DAR profiles for both ITC6103RO and ITC6104RO between the two bioanalytical methods from the in vitro linker stability and the in vivo mouse pharmacokinetic samples, respectively.

## 4. Discussion and Conclusions

With the development of the innovative ADC’s linker conjugation platform technology, the ADC containing various linkers has been dramatically developed. Accordingly, various evaluation methods have been developed for the safety and the effectiveness of the new ADC linker. In this study, two bioanalytical approaches via the LC-qTOF-MS methods were developed and successfully applied to evaluate the in vitro and in vivo linker stability and the catabolic fate of two ADCs with different linkers (the OHPAS linker and the VC-PABC linker) of ADCs with the same backbone antibody and payload. Using the “bottom-up” approach, the components of ADC were quantified and the DAR profile was calculated as the relative ratio of molar concentrations for tAb and acDrug. Using the “middle-up” approach, the intact protein mass analysis at the subunit intact protein level was assessed for the catabolic fate of each ADC. As a result, the ADC with the OHPAS linker (ITC6103RO) was stable in both mouse and IgG depleted human plasma, whereas the ADC with the VC-PABC linker (ITC6104RO) was unstable in mouse plasma. In addition, the in vivo pharmacokinetic study in mice for two ADCs also showed consistent results with the in vitro stability data which showed low exposure and high clearance of acDrug with a VC-PABC linker. The results of intact mass protein analysis showed that the VC-PABC linker of ITC6104RO is considered to be susceptible to amide hydrolysis of the PABC site by Ces1c enzyme in mouse plasma. On the other hand, the OHPAS linker of ITC6103RO was not catabolized under the same conditions.

In conclusion, the two bioanalytical approaches via the LC-qTOF-MS methods used in this study are considered to be helpful in the evaluation of the in vitro linker stability, PK properties and catabolic fate of ADCs in the early stages of development, and further experiments would be warranted to demonstrate the efficacy and toxicity related to the perspective linker.

## Figures and Tables

**Table 1:** The mass spectrometric conditions for quantification.

Scan Mode	TOF-MS Scan	Product Ion Scan			
		Specific Signature Peptide [M + 2H]^2+^	Stable Isotope-Labeled Peptide [M + 2H]^2+^	MMAF [M + H]^+^	Verapamil [M + H]^+^
Mass range (*m*/*z*)	100~1000	832~842	837~847	100~750	100~500
Parent ion (*m*/*z*)	-	937.5	942.3	732.5	455.3
Product ion (*m*/*z*)	-	836.4	842.4	700.5	165.1
Declustering potential (V)	100	162	162	100	125
Collision energy (V)	10	38	38	35	30
Accumulation time (s)	0.15	0.1	0.1	0.1	0.1

**Table 2:** The mass spectrometric conditions for intact protein mass analysis.

Scan Mode	TOF-MS Scan
Mass range (*m*/*z*)	1000~3500
Declustering potential (V)	150
Collision energy (V)	20
Accumulation time (s)	0.2

**Table 3:** Pharmacokinetic parameters of tAb after intravenous administration of 3 mg/kg of ITC6103RO and ITC6104RO in mice.

Drug	AUC_last_ (µg/day/mL)	CL (mL × day/kg)	α-HL (day)	β-HL (day)	C_max_ (µg/mL)	V_1_ (mL/kg)	V_ss_ (mL/kg)
ITC6103RO	68.00 ± 18.51	44.79 ± 14.13	0.44 ± 0.25	6.40 ± 3.48	60.41 ± 5.75	76.45 ± 7.49	236.28 ± 40.37
ITC6104RO	71.01 ± 10.13	39.40 ± 4.41	0.25 ± 0.22	4.75 ± 1.63	58.83 ± 11.60	60.95 ± 14.57	203.88 ± 48.68

AUC_last_, Area under the curve up to last measurable concentration; CL, Clearance; α-HL, Alpha phase half-life; β-HL, Beta phase half-life; Cmax, Maximum plasma concentration; V_1_, Volume of distribution of central compartment, V_ss_, Volume of distrution at steady-state.

**Table 4:** Pharmacokinetic parameters of acDrug after intravenous administration of 3 mg/kg of ITC6103RO and ITC6104RO in mice.

Drug	AUC_last_ (ng/day/mL)	CL (mL × day/kg)	α-HL (day)	β-HL (day)	C_max_ (ng/mL)	V_1_ (L/kg)	V_ss_ (L/kg)
ITC6103RO	433.30 ± 83.11	6839.58 ± 1195.49	0.17 ± 0.01	2.32 ± 0.23	585.38 ± 66.23	6.41 ± 0.96	15.97 ± 1.24
ITC6104RO	53.55 ± 0.77	55514.72 ± 693.39	0.06 ± 0.00	0.77 ± 0.20	518.61 ± 36.82	7.18 ± 0.06	10.44 ± 0.87

## Data Availability

The data presented in this study are available on request from the corresponding author.
